# Simulation and Experimental Investigation of Structural Dynamic Frequency Characteristics Control

**DOI:** 10.3390/s120404986

**Published:** 2012-04-18

**Authors:** Xingwu Zhang, Xuefeng Chen, Shangqin You, Zhengjia He, Bing Li

**Affiliations:** 1 State Key Laboratory for Manufacturing System Engineering, School of Mechanical Engineering, Xi'an Jiaotong University, Xi'an 710049, China; E-Mails: zhangxingwu1984@yahoo.com.cn (X.Z.); hzj@mail.xjtu.edu.cn (Z.H.); bli@mail.xjtu.edu.cn (B.L.); 2 System Engineering Research Institute, Beijing 100094, China; E-Mail: yshq234@126.com

**Keywords:** active control, dynamic frequency characteristics, neural network, simulation, experiment

## Abstract

In general, mechanical equipment such as cars, airplanes, and machine tools all operate with constant frequency characteristics. These constant working characteristics should be controlled if the dynamic performance of the equipment demands improvement or the dynamic characteristics is intended to change with different working conditions. Active control is a stable and beneficial method for this, but current active control methods mainly focus on vibration control for reducing the vibration amplitudes in the time domain or frequency domain. In this paper, a new method of dynamic frequency characteristics active control (DFCAC) is presented for a flat plate, which can not only accomplish vibration control but also arbitrarily change the dynamic characteristics of the equipment. The proposed DFCAC algorithm is based on a neural network including two parts of the identification implement and the controller. The effectiveness of the DFCAC method is verified by several simulation and experiments, which provide desirable results.

## 1. Introduction

Over the past several decades, structural vibration control has attracted the attention of many scholars and has led to many achievements both in theory and application. Active control has attracted much more attention in recent years because it has many attractive properties, such as flexibility and adaptability.

Balas [1,2] and Meirovitch [3] laid the foundation for the vibration control of flexible structural systems using a fully active control method. Widrow [4] achieved active motion control of an inverted pendulum by using a simple network. Chomette [5] investigated semi-adaptive modal control of on-board electronic boards using an identification method, and simulation and experimental tests indicated that this method greatly increases the nominal robustness of the controller. Song [6] adopted the adaptive fuzzy sliding mode control algorithm to actively control nonlinear structural vibration. Yue [7] studied the modal control of open cylindrical shells using non-contact actuators on the basis of photovoltaic and piezoelectric effects. Yan [8] addressed a feedback control mechanism and its optimization for rotating disk vibration/flutter via changes of air-coupled pressure generated using piezoelectric patch actuators. Radecki [9] did active vibration control in cutting tools using collocated piezoelectric sensor/actuator. Suzuki [10] demonstrated the vibration control of a flexible cantilever beam using shape memory alloys as actuators. The aforementioned studies mainly focused on time-domain vibration control. Time-domain active control methods have many advantages, such as excellent adaptability and wide application scope, but the limitations of these methods should also be considered. Time-domain active control methods should update the control parameters at every sample point, so the control algorithm should not be very complex due to the limitation of the calculation time. Secondly, time-domain active control methods commonly control a wide frequency band but they cannot focus on some major response frequency node. Time-domain active control methods are also sensitive to signal fluctuations. Furthermore, the transient response will also affect the control results, so it places high demands on the hardware equipment in practical applications.

Frequency-domain active control methods which are based on linear quasi-static assumptions can avoid the effects of transient response. They are suitable for active structural control with a periodic response. Noise and data reduction can be implemented relatively easily, and it can significantly reduce the computational complexity [11–13]. Pearson [14] compared active control in time- and frequency-domains and indicated that frequency-domain active control is an adaptive method that is suitable for vibration control in the steady-state. On the basis of the modal-free method in frequency-domain, Meurers [15] achieved active sound and vibration control. Fleischer [16] used active control methods to reduce the vibration of an electric locomotive, and a new effort-minimized equivalent modal state control concept was proposed to improve computational efficiency. Gu [17] constructed an active control algorithm in the frequency-domain on the basis of neural networks to control the vibration and noises of a rotor system. Li [18] constructed a model by using a frequency-domain identification method and applied vibration control to a flexible plate. To solve the delay in a time-domain broadband filter, Kuo [19] constructed a frequency-domain delay-less active sound quality control algorithm. Longman [20] constructed repetitive controllers in the frequency-domain for multi-input multi-output systems. Sun [21] addressed the problem of active vehicle suspension systems control in the finite frequency-domain. The aforementioned frequency-domain active control methods mainly focused on vibration and noise control but could not arbitrarily change the dynamic characteristics of the equipment for specific engineering requirements. That is, if the current dynamic frequency characteristics want to be artificially changed, the present study mentioned it less.

Therefore, a new method of dynamic frequency characteristics active control (DFCAC) is proposed first to deal with this problem. In general, mechanical equipment such as cars, airplanes, and machine tools all operate with constant frequency characteristics. These constant working characteristics should be controlled if the dynamic performance of the equipment must be improved or if the dynamic characteristics are changed to satisfy different working conditions. For example, in order to improve the stability, robustness of the equipment and the comfort to the operators, the frequency components of resonance should be avoided, interference, and some new frequency components should be added to the frequency response of the equipment to improve the stability.

Based on the frequency-domain active control methods, the error criterion is derived aiming at changing the frequency response in the frequency domain, and then the new DFCAC method is established. Compared with the aforementioned active control methods, there are some difficulties and advantages in constructing the DFCAC method. First, the objective function of the DFCAC method can be any desired frequency signal. Besides, the objective signal is a frequency signal that can reflect the frequency properties expected to the equipment. Second, the signals, the parameters and the control process are in the frequency-domain in the DFCAC method, which can save processing time associated with Fast Fourier Transform (FFT) and Inverse Fast Fourier Transform (IFFT) computation and which is convenient for control processes. A frequency error is chosen for iteration in the DFCAC method. The global error and frequency node error are combined together as the error criterion, which can improve the flexibility, adaptability and anti-jamming capability of the DFCAC method.

In Section 2, the DFCAC method is constructed using neural networks. The error criterion consisting of global and frequency node error is introduced in Section 3. In Section 4, simulations and experiments under different working conditions are implemented and the efficiency of the DFCAC method is verified.

## 2. Dynamic Frequency Characteristics Active Control System

**Figure 1.**
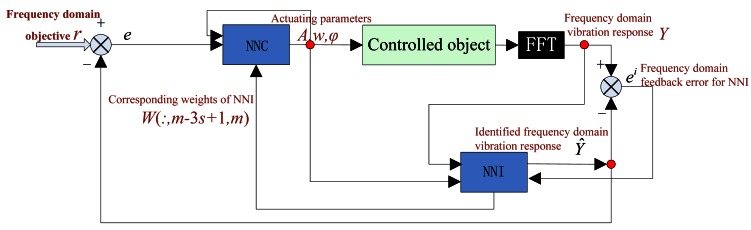
The control frame of the DFCAC system.

A neural network is constituted by many nonlinear cells and is widely used in mechanisms, aviation, banking, finance, amusement, and so forth. Neural network is very good at modeling complex and nonlinear relationships between input and output variables and is suitable for adaptive control. Hence, a neural network is chosen as the main algorithm in the DFCAC method. Figure 1 shows the system frame. The control algorithm is mainly constructed using two parts: a neural network controller (NNC) and neural network identification (NNI). The NNI identifies the system model for the controlled object, and the NNC is responsible for the control target of the whole system. First, the controlled object is excited by the actuators, where *A, w* and *φ* are the actuating amplitude, the actuating frequency and the actuating phase respectively. Then, the vibration response, which is collected by the sensors and corresponding devices, is converted to the frequency-domain by FFT. Taking the frequency domain vibration response *Y* and actuating parameters *A, w, φ* as inputs into the NNI, the frequency model of the controlled object is identified. Then, the identified frequency-domain vibration response *Ŷ* is compared with the objevtive function *r*. If the difference *e* between *Ŷ* and *r* satisfies the required precision, the control process will stop. If not, the identified model parameters *W* and *e* and actuating parameters *A, w, φ* are input into the NNC to generate new actuating parameters. This cycle is repeated until *e* satisfies the expected precision. It can be seen from the control flow, the FFT is only used once in each control cycle, and no IFFT is included, so the control process is simplified, and processing time is reduced.

**Figure 2.**
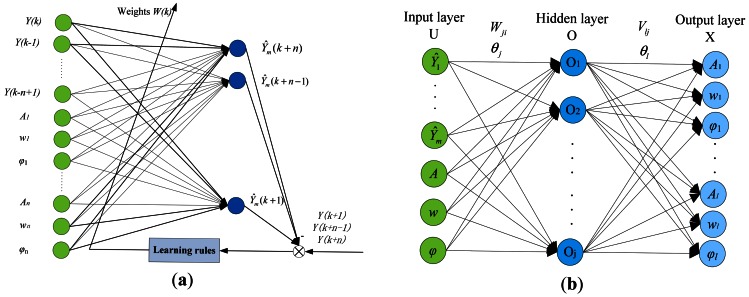
The network structures of the (**a**) NNI (**b**) NNC.

The network structures of the NNI and NNC are shown in Figure 2. The NNI is a linear network with two layers. *Y*(*k* − *n* + 1)…*Y*(*k*) are the frequency-domain vibration responses of the controlled structure. *A*_1_…*A*_n_, *w*_1_…*w*_n_ and *φ*_1_… *φ*_n_ are the actuating parameters of the actuators. *W*(*k*) represents the weights connecting input and output layers. The NNC is a nonlinear network with three layers, input layer U, hidden layer O and output layer X. *W_ji_* and *θ_j_* represent the weights and biases between the input and hidden layers. *V_lj_* and *θ_l_* are the weights and biases connecting the hidden and output layers.

In the NNI, a linear transfer function is used for the output layer. Following the Widrow-hoff principle [4], the correction of weights between the input and output of the NNI can be evaluated as:

(1) $$w_{i}(k+1)=w_{i}(k)+\frac{\alpha^{i}\cdot e^{i}(k+1)\cdot y_{i}(k)}{ɛ+y_{i}^{T}(k)\cdot y_{i}(k)}$$

where *e^i^* is the difference between the structural frequency-domain vibration response *Y* and identified output *Ŷ* of the NNI; *α^i^* ∈ (0, 2) is the attenuation factor; *ε* is a constant with a very small value that is used to avoid dividing zero when $y_{i}^{T}(k)\cdot y_{i}(k)=0$.

The network NNC has three layers, an input layer U, hidden layer O and output layer X. A tangent sigmoid transfer function is used for the hidden layer, and a linear transfer function is used for the output layer. The global frequency error *J* to be minimized by adjusting weights and biases is defined as:

(2) $$J=\frac{1}{2}\sum_{n}{\left(r_{n}-y_{n}\right)}^{2}=\frac{1}{2}{\left(r-Y\right)}^{2}$$

where *r* is the desired response of the controlled structure and *Y* represents the real-time structural frequency-domain vibration response. Using a gradient descent procedure, the correction formulas for the weights and biases connecting input and hidden layers of the NNC can be obtained by using

(3) $$W_{ji}(k+1)=W_{ji}(k)+\Delta W_{ji}$$

(4) $$\theta_{j}(k+1)=\theta_{j}(k)+\Delta \theta_{j}(k)$$

where:

$$\begin{array}{c} \Delta W_{ji}=\eta \delta_{j}.e_{i}\\ \Delta \theta_{j}=\eta \delta_{j}\\ \delta_{j}=\sum_{l}\left(r-Y).W(:,m-3s+1:m).*f\prime ({\text{net}}_{l}).V_{lj}.*f\prime ({\text{net}}_{j}\right) \end{array}$$

Here, *η* is the learning rate and represents the step size. *W*(:, *m* – 3*s* + 1:*m*) is the last 3*s* rows of the NNI weights, which represents the combination of the actuator parameters and the vibration response of the controlled plate, is the key parameters to the identified model of the control system. Here *s* is the number of the actuators. net*_j_* represents the net between input layer U and hidden layer O. net*_l_* represents the net between hidden layer O and output layer X. The correction formulas for the weights and biases between the hidden and output layers are:

(5) $$V_{lj}(k+1)=V_{lj}(k)+\Delta V_{lj}$$

(6) $$\theta_{l}(k+1)=\theta_{l}(k)+\Delta \theta_{l}(k)$$

where:

$$\begin{array}{c} \Delta V_{lj}=\eta \delta_{l}.o_{j}\\ \Delta \theta_{l}=\eta \delta_{l}\\ \delta_{l}=(r-Y).W(:,m-3s+1:m).*f\prime ({\text{net}}_{l}). \end{array}$$

To increase the learning rate and reliability, the correction formulas for the NNC based on the momentum gradient descent procedure can be obtained as follows.

For the weights and biases connecting the input and hidden layers:

(7) $$W_{ji}(k+1)=W_{ji}(k)+\Delta W_{ji}$$

(8) $$\theta_{j}(k+1)=\theta_{j}(k)+\Delta \theta_{j}(k)$$

where:

$$\begin{array}{c} \Delta W_{ji}(k)=\alpha \Delta W_{ji}(k-1)+\eta (1-\alpha )\delta_{j}.e_{i}\\ \Delta \theta_{j}(k)=\alpha \Delta \theta_{j}(k-1)+\eta (1-\alpha )\delta_{j}\\ \delta_{j}=\sum_{l}\left(r-Y).W(:,m-3s+1:m).*f\prime ({\text{net}}_{l}).V_{lj}.*f\prime ({\text{net}}_{j}\right) \end{array}$$

For the weights and biases connecting the hidden and output layers,

(9) $$V_{lj}(k+1)=V_{lj}(k)+\Delta V_{lj}$$

(10) $$\theta_{l}(k+1)=\theta_{l}(k)+\Delta \theta_{l}(k)$$

where:

$$\begin{array}{c} \Delta V_{lj}(k)=\alpha \Delta V_{lj}(k-1)+\eta (1-\alpha )\delta_{l}.o_{j}\\ \Delta \theta_{l}(k)=\alpha \Delta \theta_{l}(k-1)+\eta (1-\alpha )\delta_{l}\\ \delta_{l}=(r-Y).W(:,m-3s+1:m).*f^{\prime }({\text{net}}_{l}) \end{array}$$

Here, *η* is the learning rate and *α* is the momentum factor.

## 3. Error Criterion

As mentioned in Section 2, the global frequency error *J* in Equation (2) is chosen for each iteration in the DFCAC method. The global frequency error *J* and frequency node error *E* in Equation (11) are combined as the error criterion. The stopping criterion for each iteration is shown in Equation (12)

(11) $$E=\frac{1}{n}\sum_{n}\left(r_{k}-Y_{k}\right)$$

where *r_k_* is the desired amplitude on the *k*th characteristic frequency node. *Y_k_* is the real-time vibration amplitude on the *k*th characteristic frequency node. *n* is the number of characteristic frequency nodes:

(12) J≤err_goal1&E≤err_goal2

where err_goal1 is the convergence precision of the global frequency error, and err_goal2 is the expected precision of the frequency node error.

**Figure 3.**
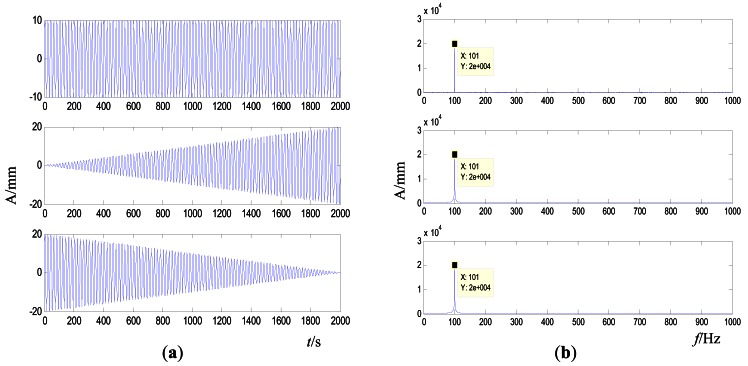
The comparison between different time- and frequency-domain signals (**a**) Time-domain signal (**b**) Frequency-domain signal.

There are some reasons and advantages for constructing this error criterion. First, constructing the frequency error *J* for each iteration can save control time and make the iterative process smooth. As shown in Equation (2), the objective *r* and frequency-domain vibration response *Y* are all frequency signals, so the frequency error optimization does not require FFT and IFFT. Furthermore, the frequency-domain signal is not in a one-to-one correspondence with time-domain signals. As shown in Figure 3, the three time-domain signals have nearly the same corresponding frequency-domain signal. Therefore, the frequency error can accurately reflect the relationship between real-time frequency vibration and the target vibration response. However, the control process may be oscillatory if the time error is chosen for each iteration in the DFCAC method.

Second, the stopping criterion for each iteration in Equation (12) can improve the flexibility, adaptability and anti-interference ability of the DFCAC method.

**Figure 4.**
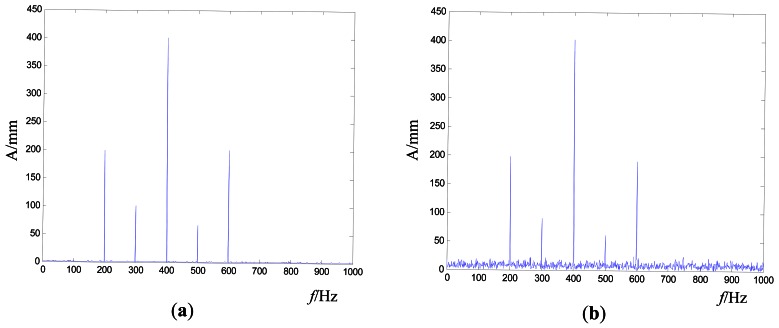
The global frequency error illustration (**a**) Target signal (**b**) Real-time vibration response.

If the global frequency error alone is taken as the stopping criterion, it may make the control process require more time or even be non-convergent. As shown in Figure 4, (a) is the target signal and (b) is the real-time vibration response. Generally, we focus on the characteristic frequency nodes. If the amplitude of these characteristic frequency nodes are close enough between the target and real-time vibration response while the amplitude of other frequency nodes are far lower, the results can be accepted, just as the results in Figure 4(b). However, the iteration cannot be stopped if the global frequency error is used as the only stopping criterion.

**Figure 5.**
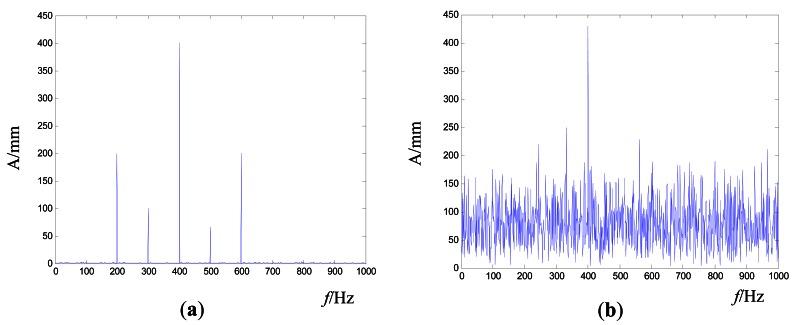
The frequency node error illustration (**a**) Target signal (**b**) Real-time vibration response.

If the frequency node error alone is taken as the stopping criterion, it may lead to some unacceptable control results. As shown in Figure 5, (a) is the target signal and (b) is the real-time vibration response. The amplitudes at 200, 300, 500 and 600 Hz are submerged by the amplitudes at other frequency nodes. Obviously, these results in Figure 5(b) cannot be accepted as the control results. However, these control processes may be stopped because the amplitudes at the frequency characteristic nodes have satisfied some target value.

Therefore, taking the global and frequency node errors together as the error criterion can avoid the two aforementioned problems. Furthermore, it can improve the flexibility, adaptability and anti-interference ability of the DFCAC method. Background noise in engineering cannot be avoided or eliminated generally, but the anti-interference ability of the DFCAC method can be improved by appropriately adjusting err_goal1 in Equation (12). Besides, the convergence rate can also be improved by adjusting err_goal1. If the background noise is within an allowable range, the convergence rate can be effectively improved by appropriately adjusting the err_goal1. Therefore, the DFCAC method has robust flexibility, adaptability and anti-interference ability when applied in practical engineering.

## 4. Simulations and Experiments

**Figure 6.**
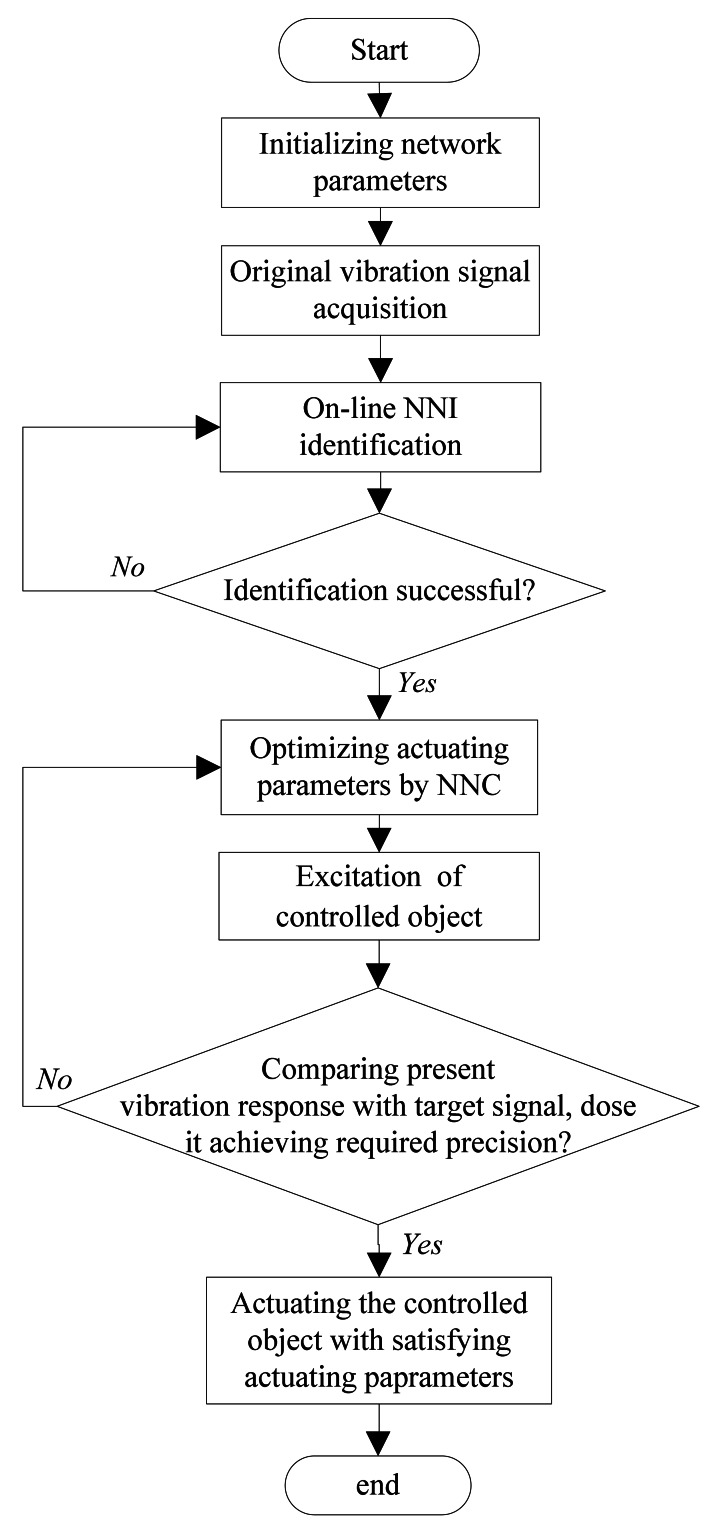
The flow chart of the DFCAC method.

The DFCAC method is implemented computationally according to the framework of the DFCAC method in Figure 1, and the frame of the NNI and NNC in Figure 2. Figure 6 shows the flowchart and some simulations and experiments of a flat plate under several different conditions are implemented following this flowchart.

### 4.1. Simulation

#### 4.1.1. Simulation System Introduction and Analysis

**Figure 7.**
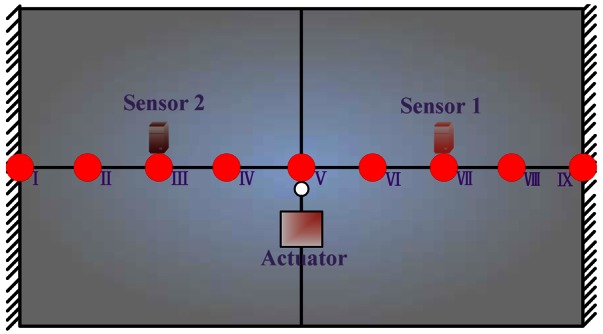
Flat plate with one actuator.

As shown in Figure 7, the simulation system mainly includes three parts: flat plate, numerical actuators and numerical sensors. The numerical sensors are simulated by a finite element method to pick the corresponding responses. The numerical actuators are simulated by adding external forces to the finite element model.

**Figure 8.**
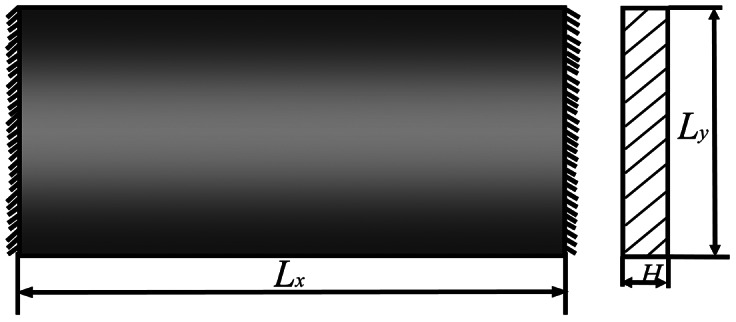
The controlled flat plate in simulation.

**Figure 9.**
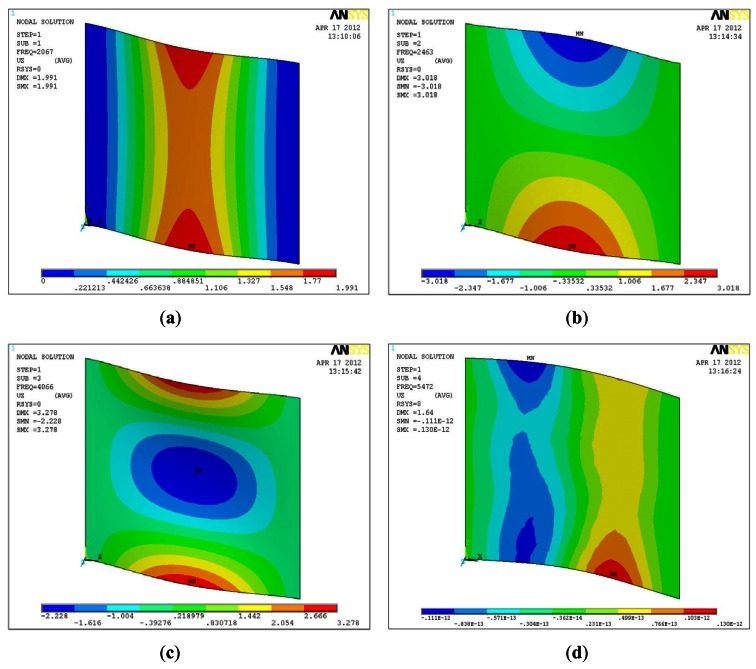

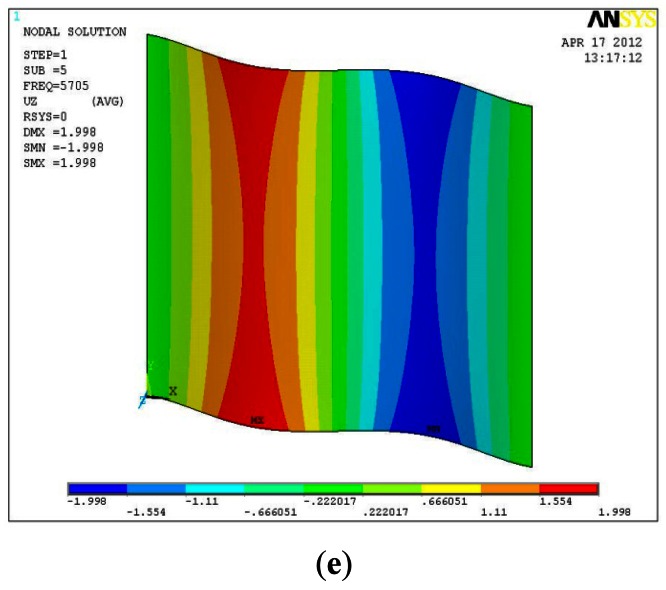
The first five mode shapes of the flat plate in simulation.

Figure 8 shows the controlled flat plate. The corresponding parameters are: plate length *L_x_* = 0.1 m, *L_y_* = 0.1 m; Elastic modulus *E* = 3.0 × 10^10^ N/m^2^; Poisson ratio *μ* = 0.3; density *ρ* = 8,000 kg/m^3^. The dynamics of the controlled flat plate is calculated by multivariable wavelet finite element method (MWFEM) [22] and the Ansys Shell63 element. The first five natural frequencies are shown in Table 1 and Figure 9 shows the first five modal shapes of the controlled plate.

#### 4.1.2. Simulation Implementation

##### Working Condition 1_Flat Plate with One Actuator

**Figure 10.**
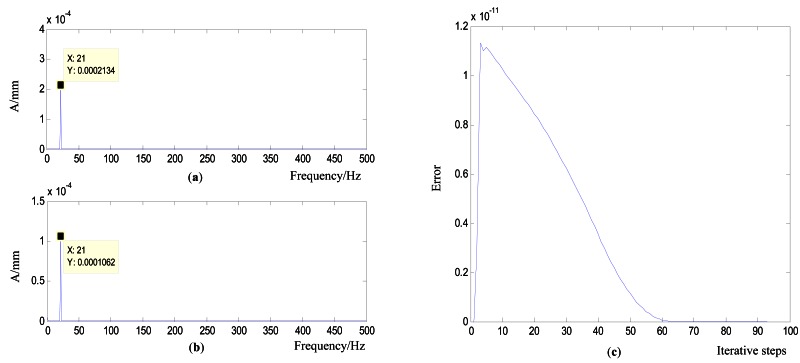
Controlled results of the simulation in working condition 1 (**a**) Original vibration bilateral spectrum (**b**) Controlled spectrum (**c**) Error curve.

As shown in Figure 7, the flat plate is excited by one actuator with variable amplitude and constant frequency. The actuators are placed according to the mode shapes of the controlled plate in Figure 9. The control objective is to reduce the value of the 21 Hz on frequency-domain to be half of its original value. Figure 10(a,b) show a comparison chart between the original bilateral spectrum and controlled spectrum. It reveals that the control successfully catches the desired response and decreases the amplitude at 21 Hz from 0.0002134 to 0.0001062 mm, exactly half of the original value. The error curve in Figure 10(c) shows that the iterative process was smooth.

##### Working Condition 2_Flat Plate with two Controlled Actuators and Two Constant Actuators

**Figure 11.**
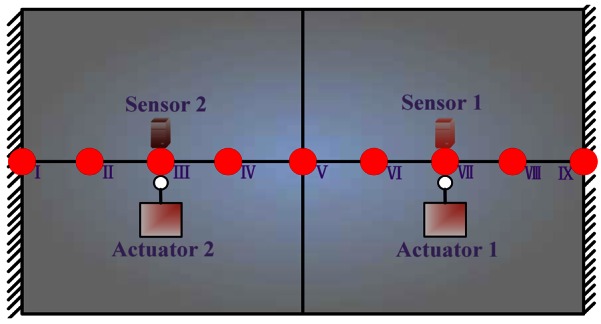
Flat plate with two actuators.

**Figure 12.**
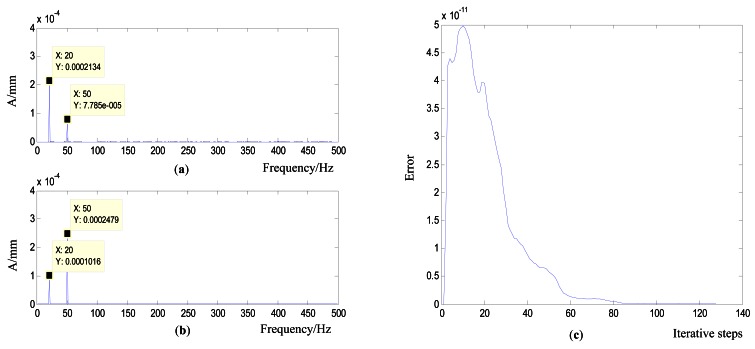
Controlled results of the simulation in working condition 2 (**a**) Original vibration bilateral spectrum (**b**) Controlled spectrum (**c**) Error curve.

As shown in Figure 11, the flat plate is originally excited by two actuators with constant parameters to simulate the disturbance and original vibrations. Actuators 1 and 2 are controlled to change the value of the constant frequency responses. The control objective was to increase the vibration response to be four times that of the original at 20 Hz and two times the original at 50 Hz. The responses collected by the two sensors, which are weighted with corresponding weights, are compared with the desired signal and feeds back the error to the NNC. The controlled results are compared in Figure 12. The results show that this control process is successful and achieves the control objective. The error curve in Figure 12 reveals that the control process was very smooth with a high convergence rate.

### 4.2. Experiments

#### 4.2.1. Experiment System Introduction and Analysis

**Figure 13.**
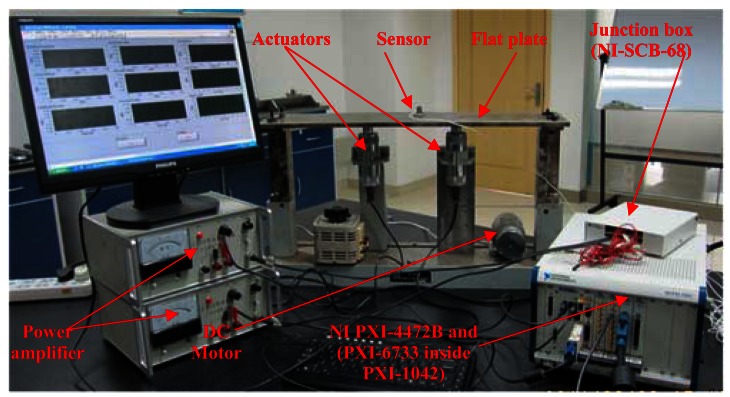
The experimental setup for the DFCAC system.

**Figure 14.**
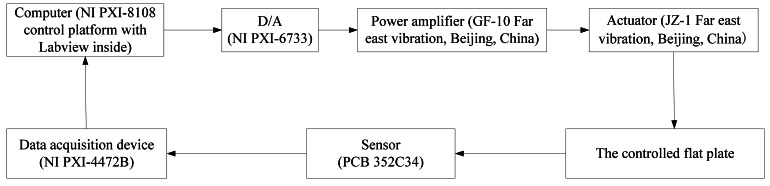
The data flow diagram and equipment model.

To verify the effectiveness of the DFCAC method in practical applications, some experimental results are discussed in this section. The experimental setup is shown in Figure 13 and the corresponding data flow diagram and equipment model are shown in Figure 14. The detailed information of the main equipments in this experiment is introduced following.

- Control platform (NI PXI-8108)
  - 2.53 GHz Intel Core 2 Duo T9400 dual-core processor
  - 1 GB (1 × 1 GB DIMM) 800 MHz DDR2 RAM standard, 4 GB maximum
  - 10/100/1000BASE-TX (gigabit) Ethernet, ExpressCard/34 slot, and 4 Hi-Speed USB ports
  - Integrated hard drive, GPIB, serial, and other peripheral I/O
- D/A (NI PXI-6733)
  - 8 high-speed digital I/O lines; two 24-bit counters; digital triggering
  - Onboard or external update clock
  - PXI trigger bus for synchronization with DAQ, motion, and vision products
- Power amplifier (GF-10 Far East Vibration, Beijing, China)
  - The maximum power: 10 W
  - The maximum current: 1 A
  - The peak voltage: 10 V
  - Frequency Range: 5 Hz–20,000 Hz
  - Size: 280 mm × 200 mm × 120 mm
- Actuator (JZ-1 Far East Vibration, Beijing, China)
  - The maximum driving force: 2N
  - Working frequency range: 5 Hz–1,000 Hz
  - The maximum displacement: ±1.5 mm
  - The maximum no-load acceleration: 67 g
  - The maximum current: 0.5 A
  - Size: Φ50 mm × 160 mm
- Sensor (PCB 352C34)
  - Sensitivity: 100 mV/g
  - Working frequency range: 0.3 Hz–15 kHz
  - Measurement range: ±50 g pk
  - Resolution: 0.00015 g
  - Temperature range: −54 °C–+93 °C
  - Size: Φ50 mm × 160 mm
- Data acquisition card (NI PXI-4472B)
  - Ability to synchronize up to 5000 channels in a PXI system
  - 24-bit resolution ADCs with 110 dB dynamic range
  - ±10 V input range or ±31 V with SMB-120 cable
  - 8 simultaneously sampled vibration-optimized analog inputs at up to 102.4 kS/s
  - Software-configurable AC/DC coupling and IEPE conditioning
- The controlled flat plate

**Figure 15.**
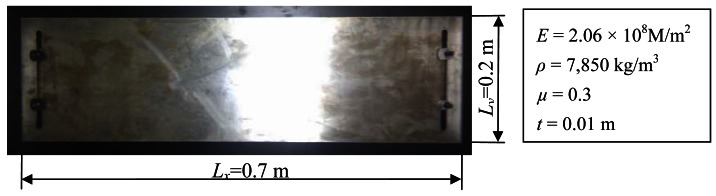
The controlled flat plate in experiment.

As shown in Figure 15, the controlled flat plate in experiment is introduced. The corresponding parameters are shown in this figure. The first three dynamic properties are analyzed by MWFEM [22], and the first three natural frequencies compared with test value are shown in Table 2.

#### 4.2.2. Experiment Implementation

According to the aforementioned experimental setup and corresponding algorithm in the simulation, the program was implemented by using LABVIEW. Several experiments under different working conditions were conducted to verify the efficiency.

**Figure 16.**
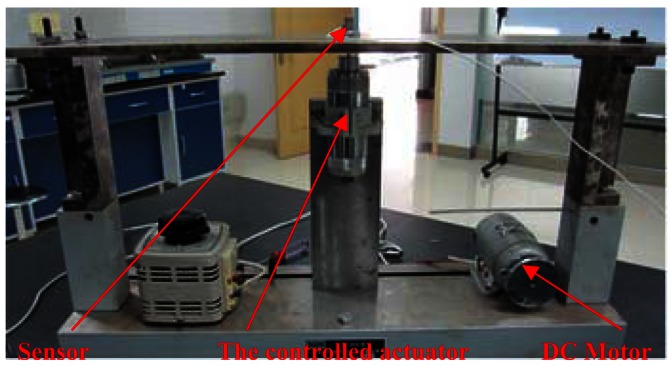
Experimental schematic diagram for working condition 1.

**Figure 17.**
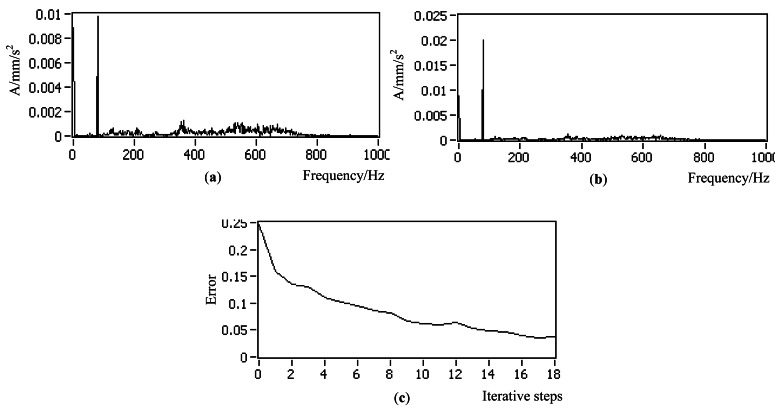
Experimental control results for working condition 1 (**a**) Original spectrum (**b**) Controlled spectrum (**c**) Error curve.

**Figure 18.**
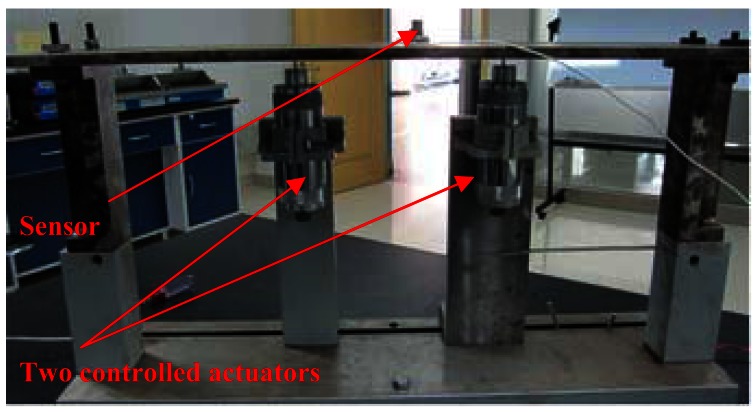
Experimental schematic diagram for working condition 2.

**Figure 19.**
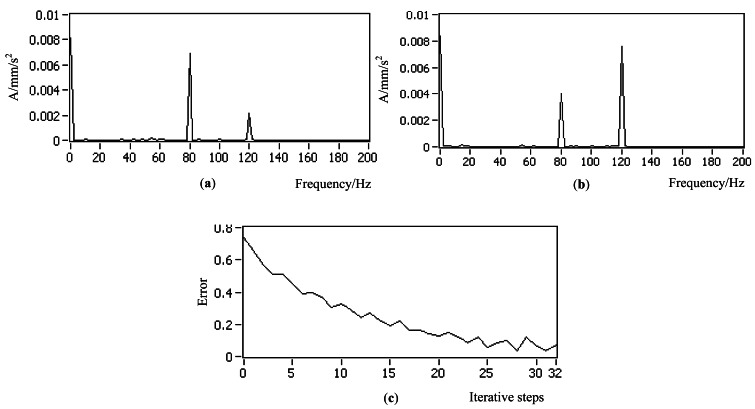
Experimental control results for working condition 2 (**a**) Original spectrum (**b**) Controlled spectrum (**c**) Error curve.

- Working condition 1_Flat plate with one controlled actuator and motor interference
- As shown in Figure 16, the flat plate was excited by one controlled actuator at central node. The DC motor acted as the interference. The control objective was to increase the vibration at 80 Hz from 0.01 to 0.02 mm. The corresponding controlled results and error curve are shown in Figure 17. The peak at 0 Hz was induced by the DC interference of the hardware device but did not affect the experimental process, which can be eliminated by some software measures or hardware method, such as removing mean in the software or adding capacitor modules in the acquisition hardware device. By comparing the controlled results, it can be seen that the control successfully achieved the objective in a noisy environment. The error curve indicates that the control process was smooth, so the anti-interference ability of the DFCAC method was also verified.
- Working condition 2_Flat plate with two controlled actuators
  As shown in Figure 18, there were two actuators in this working condition positioned at the points that are 1/4 and 3/4 length to the left end on the central line of the plate. The sensor was fitted to the central node. The control objective was to control the two actuators to change the original vibration from 0.007 to 0.004 mm at 80 Hz and 0.002 to 0.008 mm at 120 Hz in the frequency-domain. The experimental results are shown in Figure 19. By comparing the results, it is observed that this experiment achieves the control objective, and the error curve indicates that the convergence process was almost smooth.
- Working condition 3_Flat plate with one constant actuator and one controlled actuator

**Figure 20.**
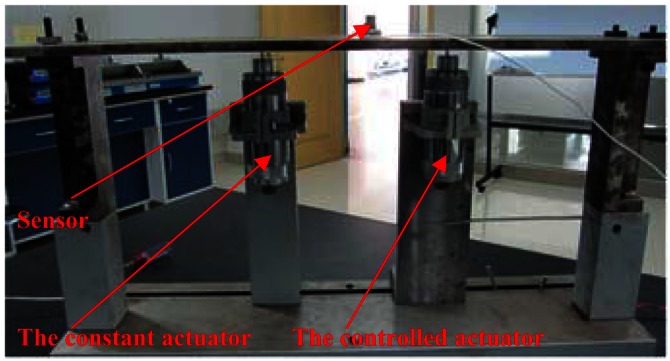
Experimental schematic diagram for working condition 3.

**Figure 21.**
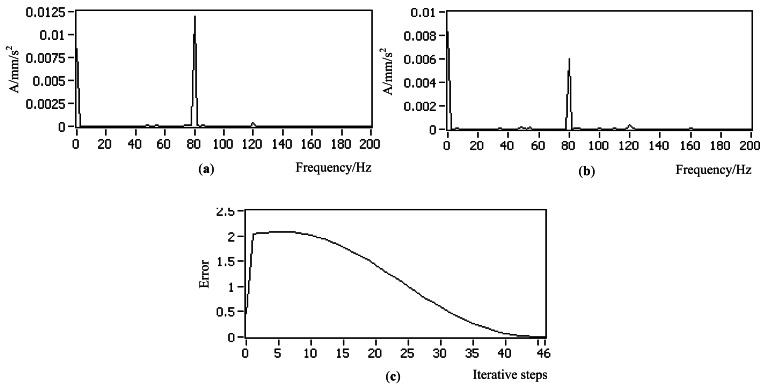
Experimental control results for working condition 3 (**a**) Original spectrum (**b**) Controlled spectrum (**c**) Error curve.

As shown in Figure 20, the controlled actuator that excited the flat plate with variable amplitude at 80 Hz was fitted to the node that is 1/4 length to the left end of the plate. The other actuator with constant amplitude was also at 80 Hz to simulate the disturbance and original vibrations. The control objective was to control the actuator to reduce the vibration response at 80 Hz from the original 0.012 to be 0.006 mm. The control results in Figure 21 indicated that the control process was stable, and the error curve shows that the convergence process was smooth.

## 5. Conclusions

On the basis of neural networks, a dynamic frequency characteristics active control method was constructed and implemented. First, neural network identification and controller systems were constructed. Then the modified formulas to the corresponding weights and biases were derived by a gradient descent procedure, and a momentum factor was brought in to improve the convergence rate. Then, an error criterion was constructed to improve the flexibility, adaptability and anti-interference ability of the method. Following that process, several simulations and experiments under different working conditions were conducted to validate the constructed algorithm. Through comparison of the control results, it can be seen that the proposed method achieves the control objective. Therefore, the DFCAC method constructed in this paper can not only achieved active vibration control, but also arbitrarily change the dynamic characteristics of mechanical devices.

## Figures and Tables

**Table 1.** The first five natural frequencies of the controlled plate in simulation.

Method(DOFs)	*f_1_*/Hz	*f_2_*/Hz	*f_3_*/Hz	*f_4_*/Hz	*f_5_*/Hz
MWFEM (484)	2,069.4	2,464.6	4,048.2	5,654.0	6,017.8
Ansys Shell63 (38400)	2,067.4	2,462.8	4,065.7	5,471.7	5,705.4

**Table 2.** The first three natural frequencies of the controlled plate in experiment.

Method	*f*_1_ (Hz)	*f*_2_ (Hz)	*f*_3_ (Hz)
MWFEM	118.86	290.68	354.81
Test	111.21	261.73	350.98
